# Slice-based YOLO-SAM hybrid prompting framework for automatic medical image segmentation

**DOI:** 10.1371/journal.pone.0360939

**Published:** 2026-11-13

**Authors:** Xiaobo Liu, Qiuyan Pei, Xiaoyu Wang, Honghang Luo

**Affiliations:** 1 Department of Nuclear Medicine, the First Affiliated Hospital, Fujian Medical University, Fuzhou, Fujian, China; 2 Department of Nuclear Medicine, National Regional Medical Center, Binhai Campus of the First Affiliated Hospital, Fujian Medical University, Fuzhou, Fujian, China; 3 College of Safety and Emergency Management Engineering, Taiyuan University of Technology, Taiyuan, Shanxi, China; 4 Nuclear Emergency and Nuclear Safety Department, China Institute for Radiation Protection, Taiyuan, Shanxi, China; 5 School of Nuclear Technology and Chemistry & Biology, Hubei University of Science and Technology, Xianning, Hubei, China; 6 Department of Economics and Management, Fujian Forestry Vocational and Technical College, Nanping, Fujian, China; National Taichung University of Science and Technology, TAIWAN

## Abstract

Accurate medical image segmentation is essential for computer-aided diagnosis, treatment planning, and quantitative image analysis. This study evaluates a slice-based automatic segmentation framework that couples YOLOv8-based region localization with prompt-driven Segment Anything Model (SAM) segmentation. In contrast to a simple YOLOv8 + SAM cascade using only bounding-box prompts, the proposed framework adds hybrid prompt generation with bounding boxes and positive/negative points, parameter-efficient fine-tuning of the SAM prompt encoder and mask decoder, and lightweight post-processing. The framework is evaluated on two public datasets under explicitly defined local protocols: 2D slice-based prostate MRI segmentation using Prostate158 and skin lesion segmentation using an internal ISIC 2018 split. On Prostate158, the method obtains Dice coefficients of 89.2%, 76.8%, and 65.3% for central gland, peripheral zone, and prostate cancer lesion segmentation, respectively. On the local ISIC 2018 split, it obtains a Dice coefficient of 94.8% and a Jaccard index of 91.5%. These results indicate competitive performance under the implemented experimental protocol, but they should not be interpreted as protocol-independent state-of-the-art claims. The manuscript clarifies the evaluation unit, prompt-source protocol, 2D processing strategy, hyperparameter settings, baseline grouping, and limitations related to statistical uncertainty, detector failure propagation, and clinical translation.

## Introduction

Medical image segmentation is one of the core tasks in medical image analysis, playing a key role in disease diagnosis, treatment planning, and prognosis evaluation [1,2]. Accurate image segmentation not only helps physicians better understand the morphological characteristics of lesions, but also provides a reliable foundation for subsequent quantitative analysis [3]. However, medical image segmentation faces numerous challenges: (1) the high complexity and diversity of medical images; (2) significant variation in the morphology, size, and location of lesion regions; (3) image quality affected by imaging equipment, parameter settings, and patient factors; and (4) difficulty and high cost of acquiring expert-annotated data [4].

Traditional medical image segmentation methods mainly rely on handcrafted features and classical machine learning algorithms, such as thresholding, region growing, and active contour models [5]. These methods often perform poorly when processing complex medical images, requiring extensive manual intervention and parameter tuning. With the rapid development of deep learning, segmentation methods based on convolutional neural networks (CNN) have gradually become mainstream [6,7]. In particular, U-Net and its variants have achieved remarkable success in medical image segmentation [8]. However, these methods typically require task-specific training, have limited generalization ability, and exhibit performance degradation in few-shot or domain-shifted scenarios.

In recent years, visual foundation models have provided new opportunities for prompt-driven segmentation [9]. The Segment Anything Model (SAM), developed by Meta AI, was trained on over one billion masks and eleven million images and supports point, box, and mask prompts [10]. Although SAM performs well in many natural-image settings, direct deployment in medical imaging remains challenging because medical images often have different gray-level distributions, modality-specific contrast, small anatomical targets, and ambiguous boundaries [11,12].

To adapt SAM to medical image segmentation, researchers have explored fine-tuning, lightweight adapters, medical-domain SAM variants, and automatic prompt generation [13,14]. A particularly practical strategy is to combine an object detector with SAM: the detector localizes regions of interest, and its output is converted into prompts for SAM [15]. Because this detection–segmentation cascade has already been reported in previous work, the present study does not claim novelty for the general idea of connecting YOLOv8 and SAM. Instead, this study focuses on the specific additions evaluated here: hybrid prompt construction, parameter-efficient SAM adaptation, and post-processing under explicitly defined medical-image protocols.

In the field of object detection, the YOLO (You Only Look Once) series is known for real-time detection performance and has been used in a range of medical imaging tasks [16–18]. In this study, YOLOv8 is used as a representative detector rather than being described as the latest or uniquely optimal detector. Its role is to generate candidate regions from which box prompts and additional point prompts are constructed.

The main contributions are as follows:

1. We formulate a slice-based automatic YOLO-SAM framework in which the main methodological additions beyond a box-only YOLOv8 + SAM baseline are hybrid positive/negative point prompts, parameter-efficient SAM fine-tuning, and lightweight mask refinement.
2. We define the prompt-source and evaluation protocols more explicitly by separating oracle-box SAM comparisons, automatic-prompt comparisons, and full end-to-end comparisons, thereby addressing the prompt-source inconsistency between GT-derived boxes and detector-predicted boxes.
3. We report results on Prostate158 and ISIC 2018 as protocol-specific benchmark evaluations rather than protocol-independent state-of-the-art claims, and we clarify the slice-based nature of the prostate MRI experiment, metric aggregation, and clinical limitations.
4. We provide reproducibility-oriented details, including modality construction, YOLO label generation, prompt hyperparameters, random sampling strategy, post-processing rules, and the additional statistical and detector-performance analyses required for an auditable experimental record.

## Related work

### Medical image segmentation

Medical image segmentation, as a core task in medical image analysis, has long been a research focus in computer vision and medical imaging. Early segmentation methods were primarily based on traditional image processing techniques, such as thresholding, edge detection, and region growing [5]. Although effective in some simple scenarios, these methods struggle to handle complex medical images.

The rise of deep learning has brought breakthrough advances in medical image segmentation. The U-Net model proposed by Ronneberger et al. [6] became the standard architecture for medical image segmentation, with its symmetric encoder-decoder structure and skip connections effectively combining high-level semantic information and low-level spatial details. Subsequently, numerous researchers improved upon U-Net, such as Attention U-Net [19], U-Net++ [20], and nnU-Net [8].

Beyond the U-Net family, other deep learning architectures have also achieved success in medical image segmentation. The DeepLab series [7,21] employs dilated convolutions and pyramid pooling to enlarge the receptive field. Transformer-based models such as TransUNet [22] and Swin-Unet [23] leverage self-attention mechanisms to capture long-range dependencies. More recently, diffusion models [24] have also been applied to medical image segmentation [25].

### Segment anything model and its medical applications

In 2023, the Segment Anything Model (SAM) [10] released by Meta AI attracted widespread attention in the computer vision community. SAM was trained on the SA-1B dataset, demonstrating strong prompt-driven segmentation capability. SAM’s architecture consists of a Vision Transformer (ViT)-based image encoder, a flexible prompt encoder, and a lightweight mask decoder. Users can provide prompts in the form of points, bounding boxes, masks, or text to guide target segmentation.

The application of SAM to medical image segmentation has rapidly become a research hotspot. Preliminary studies showed that SAM’s zero-shot performance on medical images is lower than that of specially trained models [11,12]. To improve performance, researchers have proposed various adaptation strategies:

**Fine-tuning strategies:** Ma et al. [13] proposed MedSAM, fine-tuning SAM on a large-scale medical image dataset. Wu et al. [14] proposed Medical SAM Adapter, which performs efficient adaptation by introducing lightweight modules while keeping the main SAM parameters frozen.
**Prompt engineering:** Siblini et al. [26] studied different prompting strategies for prostate segmentation and found that hybrid prompting can improve segmentation accuracy for small lesions.
**Architectural improvements:** Researchers have also proposed SAM variants, such as SAM-Med2D [27], SAM-Med3D [28], and HQ-SAM [29].

### YOLO series and its application in medical imaging

The YOLO series is a classic family of object detection algorithms, known for a favorable balance between detection accuracy and inference speed [16]. From YOLOv1 to subsequent versions, the series has evolved continuously. YOLOv8 [17] adopts an anchor-free design and improved feature extraction components.

In medical image analysis, YOLO-based detectors have been used for lesion detection, organ localization, and related tasks [30]. Examples include pulmonary nodule detection [31], cell detection [32], polyp detection [33], diabetic retinopathy detection [34], and fracture detection [35].

### Combination of YOLO and SAM

Combining detection models with segmentation models is an effective approach to realizing automated medical image analysis. Pandey et al. [15] proposed combining YOLOv8 with SAM for multimodal medical image segmentation, using YOLOv8-generated bounding boxes as prompts for SAM. Similarly, YOLOSAMIC [36] combined YOLOv8 detection with SAM-based segmentation for skin lesion analysis.

Therefore, this study avoids presenting the YOLOv8-to-SAM cascade itself as the main novelty. The technical gap addressed here is more specific: existing hybrid frameworks often use only box prompts, provide limited analysis of automatic prompt validity, and do not always separate oracle-prompt comparisons from full automatic-pipeline comparisons. The present study therefore emphasizes hybrid prompt generation, SAM decoder PEFT, post-processing, prompt-source fairness, and explicit reporting of detector failure propagation.

## Methodology

### Overall framework

As shown in Fig 1, the proposed YOLO-SAM hybrid segmentation framework consists of three main modules: the YOLOv8 detection module, the SAM segmentation module, and the post-processing module.

**Fig 1:**
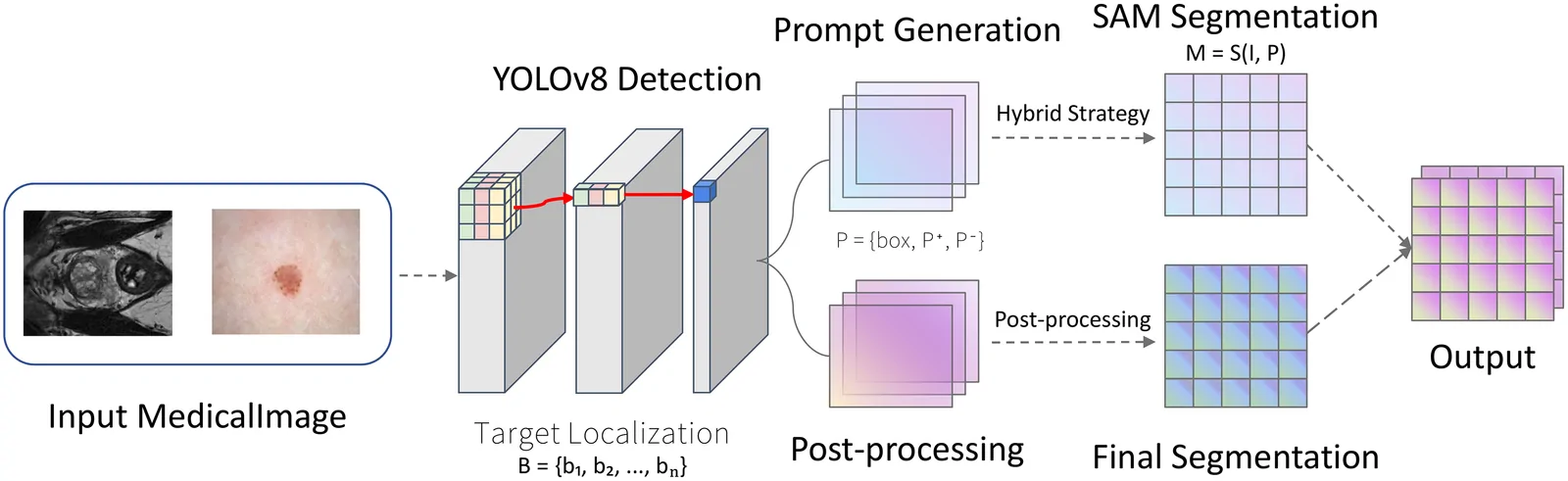
Overall architecture of the YOLO-SAM hybrid prompting framework. The detector first predicts candidate boxes. The prompt generator converts each retained detection into a box prompt plus deterministic positive and negative point prompts. The fine-tuned SAM component then produces preliminary masks, followed by thresholding, connected-component filtering, and optional morphological refinement.

An input medical image is first passed through the YOLOv8 detection module to localize regions of interest. Based on the detection results, diverse prompts are generated. The image and prompts are then fed into the fine-tuned SAM model to generate preliminary segmentation masks, which are subsequently refined by the post-processing module. Given an input image $I\in {\mathbb{R}}^{H\times W\times 3}$, the pipeline is described as follows.

**Step 1: Object Detection.** The YOLOv8 detector 𝒟 identifies target regions in the image:

$$\begin{array}{c} ℬ=\{b_{1},b_{2},...,b_{N}\}=𝒟(I), \end{array}$$ (1)

where $b_{i}=(x_{i},y_{i},w_{i},h_{i},c_{i},s_{i})$ denotes the center coordinates, width, height, category, and confidence score of the *i*-th detection box, and *N* is the number of detected objects after confidence filtering and non-maximum suppression.

**Step 2: Prompt Generation.** Based on the retained detection boxes, we generate hybrid prompts 𝒫:

$$\begin{array}{c} {𝒫}_{i}=\text{PromptGen}(b_{i},I)=\{box_{i},P_{i}^{+},P_{i}^{-}\}, \end{array}$$ (2)

where $box_{i}$ is the bounding box prompt, and $P_{i}^{+}$ and $P_{i}^{-}$ are the positive and negative point sets, respectively.

**Step 3: Segmentation Generation.** The image and prompts are fed into the fine-tuned SAM model 𝒮:

$$\begin{array}{c} M_{i}=𝒮(I,{𝒫}_{i}), \end{array}$$ (3)

where $M_{i}\in [0,1{]}^{H\times W}$ is the probability mask for the *i*-th target.

**Step 4: Post-processing.** Post-processing operations ℛ are applied to refine and binarize the segmentation result:

$$\begin{array}{c} {\hat{M}}_{i}=ℛ(M_{i};\tau ,A_{\min},k_{1},k_{2}), \end{array}$$ (4)

where τ is the mask threshold, $A_{\min}$ is the minimum connected-component area, and *k*_1_ and *k*_2_ are structuring-element sizes.

If no detection box is produced for a target class, the end-to-end prediction for that class is scored as an empty mask. This rule keeps detector failures in the final segmentation score and prevents conditional reporting after successful localization only. Conditional results after successful detection are reported separately only to isolate the SAM segmentation stage from detector failure.

### Modality selection and label construction

For Prostate158, all slices from the same patient are kept within the same train, validation, or test partition to avoid patient-level leakage. The experiment is explicitly slice-based. For anatomical zone segmentation, the T2-weighted sequence is used as the primary input because it provides the clearest zonal anatomical contrast. For PCa lesion segmentation, the multiparametric input is constructed from registered T2W, DWI, and ADC images when all three sequences are available; otherwise, the missing-channel handling rule is stated in the released preprocessing script. All modalities are resampled to a common in-plane resolution and aligned before conversion to a three-channel tensor expected by YOLOv8 and SAM.

YOLO detection labels are generated from the segmentation masks by computing the smallest axis-aligned rectangle enclosing each non-empty connected target mask at the slice level. Empty masks are retained for segmentation metric aggregation but do not generate detector boxes. For slices with multiple lesions, one detection label is generated for each connected lesion component. When multiple YOLO boxes overlap after inference, non-maximum suppression is applied within each class. When predicted masks of CG, PZ, and PCa overlap, class-specific masks are evaluated independently because the clinical targets are not mutually identical anatomical categories.

For ISIC 2018, RGB dermoscopy images are resized and normalized before detection and segmentation. The exact training, validation, and test image identifiers, together with the random seed used for the internal split, are treated as supporting materials so that the local split remains auditable.

### YOLOv8 detection module

We select YOLOv8 as the detection backbone because it provides a practical balance between detection accuracy and inference speed. For the Prostate158 dataset, we train YOLOv8 to detect three target categories: central gland (CG), peripheral zone (PZ), and prostate cancer lesions (PCa). For the ISIC 2018 dataset, the detection target is the skin lesion region.

The training process uses an input size of 640×640, batch size of 16, AdamW optimizer with learning rate 10^–3^, CIoU loss combined with binary cross-entropy classification loss, and data augmentation including random rotation ($\pm 10^{\circ }$), horizontal flip, and color jitter.

To make the detection stage reproducible, the detector model size, initialization strategy, training schedule, confidence threshold, and non-maximum suppression setting are reported together with the segmentation settings in Table 1. The same detector configuration is used for detector-only evaluation and full end-to-end segmentation evaluation.

**Table 1:** Reproducibility-oriented hyperparameter checklist for the experiments.

Item	Prostate158	ISIC 2018
YOLOv8 model size	YOLOv8m	YOLOv8s
YOLO initialization	COCO-pretrained yolov8m.pt	COCO-pretrained yolov8s.pt
Detector input size	640×640	640×640
Detector batch size	16	16
Detector learning rate	10^–3^	10^–3^
Detector epochs / early stopping	96 / patience 18	112 / patience 22
Confidence threshold	CG 0.27; PZ 0.24; PCa 0.16	0.23
NMS IoU threshold	0.52	0.47
*T*_medium_, *T*_large_	1,024 / 9,216 pixels	9,216 / 65,536 pixels
Negative points *n*_–_	2	2
Safety distance δ	8 pixels	16 pixels
SAM backbone/checkpoint	ViT-B / sam_vit_b_01ec64.pth	ViT-B / sam_vit_b_01ec64.pth
Frozen / trainable modules	Image encoder frozen; prompt encoder and mask decoder trainable	Image encoder frozen; prompt encoder and mask decoder trainable
Total / trainable parameters	93.7M / 4.32M	93.7M / 4.32M
SAM learning rate	10^–4^	10^–4^
SAM epochs / early stopping	50 / patience 10	50 / patience 10
Mask threshold τ	0.52	0.48
$A_{\min}$	64 pixels	256 pixels
Random seed	17821	50937

### Hybrid prompt generation strategy

The quality of prompts directly affects SAM’s segmentation performance. We design a hybrid prompt generation strategy that combines bounding box and point prompts.

**Bounding box prompts:** The retained detection boxes from YOLOv8 are directly used as bounding box prompts, providing SAM with coarse location information about the target.

**Positive point prompts:** Positive points are sampled inside the detection box to guide SAM to focus on internal target features. The sampling strategy is as follows:


$$\begin{array}{c} P^{+}=\{p_{j}|p_{j}=(x_{c}+\Delta x_{j},\;y_{c}+\Delta y_{j}),\;j=1,...,n_{+}\}, \end{array}$$ (5)

where $\left(x_{c},y_{c}\right)$ is the center of the detection box, and $\left(\Delta x_{j},\Delta y_{j}\right)$ are offsets satisfying $|\Delta x_{j}|<w/4$ and $|\Delta y_{j}|<h/4$. The number of positive points *n*_+_ is adaptively adjusted based on target size:


$$\begin{array}{c} n_{+}=\left\{ \begin{array}{cc} 1 & \text{if }A>T_{\text{large}},\\ 2 & \text{if }T_{\text{medium}}<A\leq T_{\text{large}},\\ 3 & \text{if }A\leq T_{\text{medium}}, \end{array}\right. \end{array}$$ (6)

where A=w×h is the detection box area.

Because a point sampled from the central part of a predicted box is not guaranteed to lie inside an irregular target, these prompts are interpreted as detector-derived heuristic positive prompts rather than ground-truth-verified positive labels at inference. To audit this assumption, the evaluation records, retrospectively on the annotated test set, the proportion of generated positive points falling outside the reference mask. Inference uses a fixed random seed or a deterministic grid-based sampling rule; if random sampling is retained, repeated prompt draws are evaluated to estimate inference variability.

**Negative point prompts:** Negative points are sampled outside the detection box to help SAM distinguish the target from surrounding background:


$$\begin{array}{c} P^{-}=\{p_{k}|d(p_{k},box)>\delta ,\;k=1,...,n_{-}\}, \end{array}$$ (7)

where $d(p_{k},box)$ denotes the distance from point $p_{k}$ to the bounding box, δ is a safety-distance threshold, and *n*_–_ is the number of negative points.

Compared with a box-only prompt, the hybrid prompt is intended to provide additional foreground and background cues. Performance gains are therefore attributed specifically to box-plus-point prompting only when the comparison is conducted under the same detector, data split, SAM checkpoint, post-processing rule, and evaluation unit.

### SAM fine-tuning strategy

Although SAM possesses strong prompt-driven segmentation capability, a performance gap remains when applied directly to medical images. We adopt a Parameter-Efficient Fine-Tuning (PEFT) strategy to adapt SAM.

**Fine-tuning architecture:** We freeze the SAM image encoder and fine-tune the prompt encoder and mask decoder. This strategy preserves the universal visual representation learned by the image encoder while allowing the prompt-conditioned segmentation head to adapt to medical-image characteristics.

The SAM backbone, checkpoint, frozen modules, trainable modules, total parameter count, and trainable parameter count are listed with the other implementation settings in Table 1.

**Loss function:** We adopt a hybrid loss function combining Dice loss and binary focal loss:


$$\begin{array}{c} ℒ=\lambda_{1}{ℒ}_{\text{Dice}}+\lambda_{2}{ℒ}_{\text{Focal}}. \end{array}$$ (8)

The Dice loss is defined as:


$$\begin{array}{c} {ℒ}_{\text{Dice}}=1-\frac{2\sum_{i=1}^{HW}p_{i}g_{i}+\epsilon }{\sum_{i=1}^{HW}p_{i}+\sum_{i=1}^{HW}g_{i}+\epsilon }, \end{array}$$ (9)

where $p_{i}$ is the predicted probability, $g_{i}$ is the ground-truth label, and ϵ=1 is a smoothing term.

The implemented binary focal loss includes both foreground and background terms:


$$\begin{array}{c} {ℒ}_{\text{Focal}}=-\frac{1}{HW}\sum_{i=1}^{HW}\left[\alpha g_{i}(1-p_{i}{)}^{\gamma }\log(p_{i}+\varepsilon )+(1-\alpha )(1-g_{i})p_{i}^{\gamma }\log(1-p_{i}+\varepsilon )\right]. \end{array}$$ (10)

Here, α is the foreground weight, γ=2 is the focusing parameter, and ε is used for numerical stability. In our experiments, $\lambda_{1}=0.5$ and $\lambda_{2}=0.5$.

The training configuration uses a learning rate of 10^–4^ with cosine annealing schedule, AdamW optimizer with weight decay 10^–4^, batch size of 4, 50 training epochs, and early stopping with patience = 10.

### Post-processing module

To refine the segmentation results, we design a post-processing module consisting of thresholding, morphological operations, connected-component filtering, and optional boundary smoothing.

**Thresholding:** The probability mask is first converted into a binary mask:


$$\begin{array}{c} M_{b}=𝕀(M\geq \tau ). \end{array}$$ (11)

**Morphological operations:** Opening is used to remove small noise regions, and closing is used to fill small holes:


$$\begin{array}{c} M^{\prime }=\text{Close}(\text{Open}(M_{b},k_{1}),k_{2}), \end{array}$$ (12)

where *k*_1_ and *k*_2_ are the sizes of the structuring elements.

**Connected component filtering:** Connected components with an area smaller than $A_{\min}$ are removed:


$$\begin{array}{c} \tilde{M}=⋃\{C\in \text{ConnectedComponents}(M^{\prime })|\text{Area}(C)>A_{\min}\}. \end{array}$$ (13)

This equation retains all components above the area threshold. When only the largest component is retained for a specific task, that task-specific rule is explicitly stated.

**Boundary smoothing and re-binarization:** Gaussian filtering is applied to smooth the binary mask, followed by re-binarization:


$$\begin{array}{c} \hat{M}=𝕀(\text{GaussianFilter}(\tilde{M},\;\sigma =1.0)\geq 0.5). \end{array}$$ (14)

### Hyperparameter and reproducibility details

The implementation settings are consolidated in Table 1, including detector training, prompt construction, SAM adaptation, post-processing, and random-seed configurations for both datasets.

## Experimental setup

### Datasets

To evaluate the proposed framework, we conduct experiments on two publicly available medical image segmentation benchmarks that differ in imaging modality, anatomical target, and clinical context. The two datasets are treated as separate benchmark evaluations under their own protocols. They demonstrate multi-dataset applicability but are not used to claim protocol-independent cross-domain generalization.

#### Prostate158 dataset.

The Prostate158 dataset [37] is an expert-annotated benchmark for prostate MRI segmentation, publicly released by Charité – Universitätsmedizin Berlin. Each examination comprises a T2-weighted (T2W) sequence and diffusion-weighted imaging (DWI) together with its apparent diffusion coefficient (ADC) map. The T2W sequence provides anatomical contrast for delineating the prostate and sub-zones, whereas DWI/ADC sequences support lesion characterization.

The dataset provides annotations for three clinically relevant targets: (1) the **central gland** (CG), (2) the **peripheral zone** (PZ), and (3) **prostate cancer lesions** (PCa) with PI-RADS score ≥4.

The key characteristics of the dataset are as follows:

- **Total samples:** 158 multi-parametric prostate MRI examinations
- **Official training / test split:** 139 / 19 cases
- **Annotation targets:** CG, PZ, and PCa lesions
- **Image resolution:** resampled to 512×512 pixels in-plane
- **Slice thickness:** 3.0 mm

In our experiments, the 139 official training cases are further divided into a model training subset and a validation subset at the patient level. All slices from the same patient remain within a single partition. Metrics are first computed at the slice level for non-empty and empty masks according to the rules in the Evaluation Metrics section and are then aggregated by case to avoid over-weighting cases with more slices. The numbers of cases, total slices, positive slices, lesions, and empty masks for CG, PZ, and PCa are part of the auditable experimental record.

#### ISIC 2018 dataset.

The ISIC 2018 dataset [38], released by the International Skin Imaging Collaboration (ISIC), is a widely adopted benchmark for skin lesion analysis. The dataset contains 2,594 dermoscopy images paired with pixel-level binary lesion masks. Images include practical challenges such as hair artifacts, specular highlights, non-uniform illumination, irregular pigmentation, and ambiguous lesion boundaries.

The key characteristics of the dataset are as follows:

- **Total samples:** 2,594 dermoscopy images with paired binary segmentation masks
- **Image resolution:** variable; typical range from 540×722 to 4,499×6,748 pixels
- **Annotation quality:** pixel-level lesion boundaries
- **Clinical challenges:** hair artifacts, specular highlights, uneven illumination, blurry and irregular lesion boundaries

The reported results use an internal 70/15/15 training/validation/test split. This split is valid for comparing locally implemented methods under the same protocol, but it is not directly comparable with results reported on the official ISIC challenge test set or other private splits. Therefore, literature results under different splits are described only as contextual and non-like-for-like comparisons. The exact image identifiers and random seed for the internal split are treated as necessary metadata for reproducing the experiment.

### Evaluation metrics and aggregation protocol

The evaluation unit is explicitly defined before metric aggregation. For Prostate158, predictions are generated for 2D slices, and slice-level masks are grouped by patient to produce case-level summaries. For ISIC 2018, each dermoscopy image is one evaluation unit. Unless otherwise stated, Dice, Jaccard, sensitivity, specificity, HD, and ASD are first calculated per evaluation unit and then summarized as mean ± standard deviation. Confidence intervals are computed at the case/image level.

Empty-reference cases are handled as follows. If both the reference and prediction are empty, Dice and Jaccard are assigned 1 and the case is counted as a true empty prediction. If the reference is non-empty and the prediction is empty because YOLO misses the target, Dice and Jaccard are assigned 0, sensitivity is 0, and the error is included in the unconditional end-to-end score. If the reference is empty and the prediction is non-empty, Dice and Jaccard are assigned 0 and the result is counted as a false positive. Multiple lesions and multiple detections are evaluated after class-wise mask union unless lesion-level detection analysis is specifically reported.

**Dice Similarity Coefficient (DSC)** measures the spatial overlap between the predicted segmentation *P* and the ground-truth mask *G*:


$$\begin{array}{c} \text{DSC}=\frac{2|P\cap G|}{\left|P|+|G\right|}. \end{array}$$ (15)

**Jaccard Index (JI)** measures the ratio of the intersection to the union of the predicted and ground-truth regions:


$$\begin{array}{c} \text{JI}=\frac{\left|P\cap G\right|}{\left|P\cup G\right|}. \end{array}$$ (16)

**Hausdorff Distance (HD)** evaluates maximum boundary discrepancy:


$$\begin{array}{c} \text{HD}(P,G)=\max\left\{\underset{p\in \partial P}{\sup}\underset{g\in \partial G}{\inf}d(p,g),\;\underset{g\in \partial G}{\sup}\underset{p\in \partial P}{\inf}d(g,p)\right\}. \end{array}$$ (17)

For Prostate158, boundary distances are converted to millimetres using the image spacing after resampling. Because maximum HD is sensitive to outliers, the boundary-error interpretation is based on both HD and ASD, and robust boundary metrics such as HD95 or Surface Dice can be included in the extended statistical report.

**Average Surface Distance (ASD)** computes the mean boundary deviation:


$$\begin{array}{c} \text{ASD}(P,G)=\frac{1}{\left|\partial P|+|\partial G\right|}\left(\sum_{p\in \partial P}d(p,\;\partial G)+\sum_{g\in \partial G}d(g,\;\partial P)\right). \end{array}$$ (18)

**Sensitivity** and **Specificity** evaluate the model’s ability to correctly identify foreground and background pixels:


$$\begin{array}{c} \text{Sensitivity}=\frac{TP}{TP+FN},\;\text{Specificity}=\frac{TN}{TN+FP}. \end{array}$$ (19)

### Statistical analysis

To address statistical uncertainty, locally implemented methods are evaluated at the case or image level, and paired comparisons are conducted between the proposed method and the strongest baselines, especially MedSAM and the YOLOv8 + SAM box-only baseline. For normally distributed paired differences, a paired *t* test is used; otherwise, the Wilcoxon signed-rank test is preferred. Ninety-five percent confidence intervals are computed by non-parametric bootstrap resampling over cases or images. The word “significant” is used only when a statistical test and *p* value are reported.

### Implementation details

**Hardware configuration.** All experiments are conducted on a high-performance computing server equipped with a single NVIDIA A100 GPU (40 GB HBM2 memory) and an Intel Xeon Platinum 8380 CPU (40 cores, 2.3 GHz), running Ubuntu 20.04 LTS. All models are trained and evaluated on the same hardware environment to ensure comparable computational measurement.

**Software environment.** The implementation is built upon PyTorch 2.0.1, Python 3.9, MONAI 1.2.0, Ultralytics YOLOv8 version 8.0.196, and the official Segment Anything Model repository. Mixed-precision training (FP16) is enabled via PyTorch AMP.

**Data preprocessing.** For Prostate158, MRI slices are resampled to 512×512 pixels using bilinear interpolation, and slice-level intensity values are normalized to the [0, 1] range using per-volume minimum and maximum intensities. For ISIC 2018, images are resized to 1,024×1,024 pixels, black borders introduced by dermoscopy instrument vignetting are detected and removed via morphological operations, and pixel intensities are normalized channel-wise using ImageNet statistics.

**Inference benchmarking.** Inference time is reported separately for preprocessing, YOLO detection, prompt generation, SAM inference, and post-processing. Benchmarking uses batch size 1, includes data transfer and preprocessing unless otherwise stated, discards warm-up iterations, and reports mean ± standard deviation over repeated runs. For Prostate158, both per-slice time and approximate per-volume time are reported, because the framework processes 2D slices rather than a whole volume in one pass.

### Comparison methods

To ensure fair evaluation, comparison methods are grouped by prompt-source and automation protocol:

1. **Fully supervised segmentation baselines:** U-Net [6], nnU-Net [8], and TransUNet [22]. These methods directly output segmentation masks without SAM prompts.
2. **Oracle-prompt SAM baselines:** SAM zero-shot [10] and MedSAM [13] evaluated with ground-truth-derived boxes. These results isolate prompt-conditioned SAM segmentation ability under oracle localization and are not treated as fully automatic pipeline comparisons.
3. **Automatic-prompt SAM baselines:** YOLOv8 + SAM using predicted boxes only, and the proposed YOLOv8 + SAM using predicted boxes plus positive and negative points. These methods share the same detector outputs when used to isolate the contribution of prompt construction.
4. **Full end-to-end evaluation:** Detector failures are retained as empty predictions. Unconditional end-to-end scores are the primary automatic-pipeline results; conditional scores after successful detection are reported only as a diagnostic analysis.

## Experimental results

### Detector performance and failure propagation

Because missed detections in the YOLO stage directly propagate to the downstream SAM segmentation stage, detector performance is reported independently from segmentation performance. Table 2 records localization accuracy and the missed-detection rate for each dataset and target class.

**Table 2:** Detector performance and cascade-failure reporting structure.

Dataset / class	AP50	AP50–95	Precision	Recall	Missed-detection rate
Prostate158 / CG	0.956	0.713	0.918	0.943	5.7%
Prostate158 / PZ	0.927	0.682	0.904	0.911	8.9%
Prostate158 / PCa	0.814	0.486	0.772	0.738	26.2%
ISIC 2018 / lesion	0.972	0.831	0.948	0.961	3.9%

### Results on Prostate158 dataset

The Prostate158 experiment evaluates both anatomical-zone segmentation and prostate cancer lesion segmentation under the local slice-based protocol. Table 3 reports the central gland and peripheral zone results, with Dice, HD, and ASD used to describe overlap accuracy and boundary error. These point estimates are interpreted within the implemented protocol and are considered together with case-level uncertainty before clinical claims are made.

**Table 3:** Comparison of segmentation performance on the Prostate158 test set. Values are reported under the local slice-based protocol; Dice reflects region overlap, whereas HD and ASD describe boundary discrepancies.

Method	Central Gland			Peripheral Zone		
	DSC	HD	ASD	DSC	HD	ASD
U-Net	85.3	21.8	3.2	71.5	30.4	2.8
nnU-Net	87.1	18.5	2.7	73.8	26.1	2.1
TransUNet	86.5	19.3	2.9	72.6	28.3	2.5
SAM (zero-shot, oracle box)	78.2	28.6	4.5	65.3	38.2	3.9
MedSAM (oracle box)	87.8	17.9	2.5	74.8	25.2	2.0
YOLOv8 + SAM (automatic box only)	87.4	18.2	2.6	74.3	26.8	2.2
**Ours (automatic hybrid prompt)**	**89.2**	**16.3**	**2.1**	**76.8**	**23.5**	**1.8**

The prostate segmentation results show that the proposed automatic hybrid-prompt pipeline achieves higher point estimates than the local automatic box-only YOLOv8 + SAM baseline in Table 4. The improvement is most pronounced for PCa lesions, where small target size makes prompt quality important. However, because the Prostate158 test set contains only 19 cases, these point estimates require case-level uncertainty intervals and paired statistical tests before claims of statistically reliable superiority are made.

**Table 4:** Comparison of prostate cancer lesion segmentation performance. The results include detector-stage failure propagation in the unconditional end-to-end evaluation, while conditional results after successful localization can be used as a separate diagnostic analysis.

Method	DSC (%)	HD (mm)	Sensitivity (%)	Specificity (%)
U-Net	54.7	43.2	62.3	98.5
nnU-Net	59.8	38.5	67.1	98.9
TransUNet	57.2	41.6	64.8	98.7
SAM (zero-shot, oracle box)	45.3	52.8	53.2	97.8
MedSAM (oracle box)	61.5	36.2	68.9	99.1
YOLOv8 + SAM (automatic box only)	61.2	37.1	68.5	99.0
**Ours (automatic hybrid prompt)**	**65.3**	**32.8**	**72.6**	**99.3**

### Results on ISIC 2018 dataset

The ISIC 2018 experiment assesses whether the same automatic hybrid-prompt strategy transfers to RGB dermoscopy images. Table 5 reports overlap-, pixel-, and class-balance-oriented metrics on the internal test split, enabling comparison among locally implemented methods evaluated under the same preprocessing and data partition.

**Table 5:** Comparison of segmentation performance on the internal ISIC 2018 split. Values are comparable among locally implemented methods evaluated on the same split and preprocessing pipeline; cross-study comparison requires matched data partitions and evaluation rules.

Method	DSC (%)	JI (%)	Accuracy (%)	Sensitivity (%)	Specificity (%)
U-Net	91.2	85.6	95.3	89.7	97.8
nnU-Net	92.5	87.4	96.1	91.2	98.2
TransUNet	91.8	86.5	95.7	90.3	97.9
DeepLabV3+	92.1	87.0	95.9	90.8	98.0
SAM (zero-shot, oracle box)	86.5	79.2	93.1	83.6	96.5
MedSAM (oracle box)	93.2	88.5	96.5	92.1	98.4
YOLOv8 + SAM (automatic box only)	93.8	89.8	96.9	92.8	98.6
**Ours (automatic hybrid prompt)**	**94.8**	**91.5**	**97.6**	**94.3**	**98.9**

On the internal ISIC 2018 split, the proposed automatic hybrid-prompt method obtains higher point estimates than the locally implemented baselines. This result is interpreted as a protocol-specific improvement rather than a state-of-the-art claim, because literature protocols, data splits, preprocessing pipelines, and statistical uncertainty are not demonstrably identical.

### Ablation study

The ablation study is conducted on one fixed validation set, so all configurations are compared under the same data partition and evaluation protocol in Table 6.

**Table 6:** Ablation study on the fixed Prostate158 validation set. All rows are evaluated on the same validation split under the same protocol.

Configuration	CG DSC (%)	PZ DSC (%)	Lesion DSC (%)
Box prompt only	87.1	73.8	60.5
+ Positive point prompts	88.3	75.2	62.8
+ Negative point prompts	88.9	76.1	64.2
+ Adaptive point count	89.0	76.5	64.9
+ SAM fine-tuning	89.5	77.2	65.8
+ Post-processing	**89.8**	**77.6**	**66.2**

The validation ablation suggests incremental gains when positive points, negative points, adaptive point counts, SAM fine-tuning, and post-processing are added. The lesion Dice increase from 60.5% to 66.2% reflects the combined contribution of all added components, not hybrid prompting alone. The prompt-only contribution is interpreted from the controlled prompt comparison in Table 7.

**Table 7:** Performance comparison of different prompt types on prostate lesion segmentation.

Prompt Type	DSC (%)	HD (mm)	Sensitivity (%)
Box only	60.5	38.2	67.3
Point only (3 points)	58.2	41.5	64.8
Box + 1 positive point	62.8	36.1	69.5
Box + 2 positive points	64.1	34.8	71.2
Box + 3 positive points	64.3	34.5	71.5
Box + positive + negative points	**65.3**	**32.8**	**72.6**

### Comparison of different prompt types

To isolate the effect of prompt design, prostate lesion segmentation is evaluated under several prompt combinations while keeping the remaining pipeline unchanged. Table 7 shows how box prompts, positive points, and negative points affect overlap accuracy, boundary error, and sensitivity.

Compared with box-only prompting, adding positive and negative points improves lesion Dice from 60.5% to 65.3%, corresponding to a 4.8-percentage-point increase under this protocol. The gain is therefore attributed to the controlled box-plus-point prompt comparison.

### Visualization analysis

Fig 2 presents qualitative examples.

**Fig 2:**
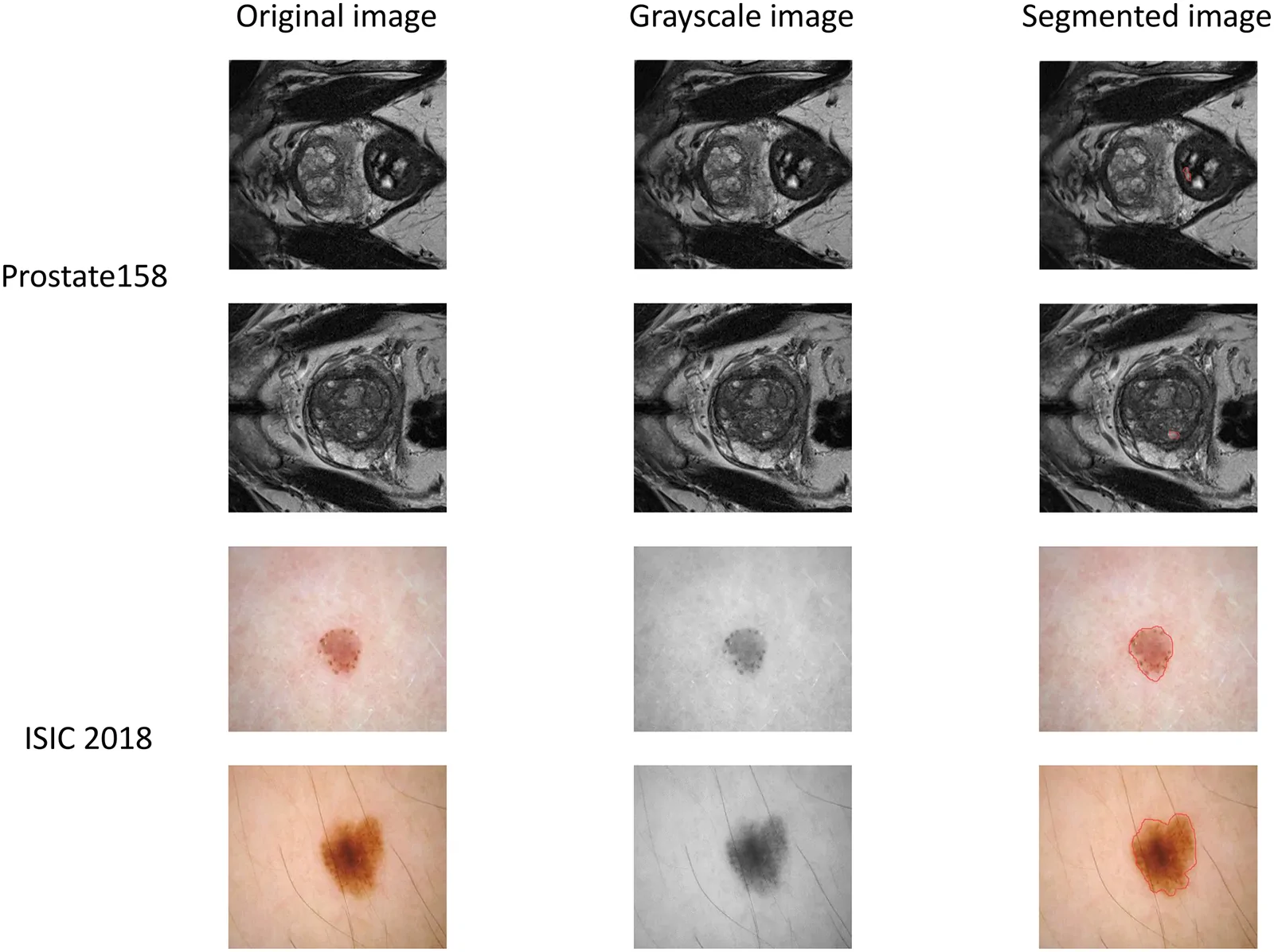
Visualization examples. From left to right, the panels show the original image, the grayscale or preprocessed image, and the segmentation output generated by the proposed pipeline.

The qualitative examples are used only as visual support. Claims about systematic robustness to small lesions or boundary-ambiguous regions require size-stratified, boundary-difficulty-stratified, or uncertainty-aware quantitative analyses and are therefore not stated as established conclusions.

Fig 3 illustrates the training dynamics of the proposed method.

**Fig 3:**
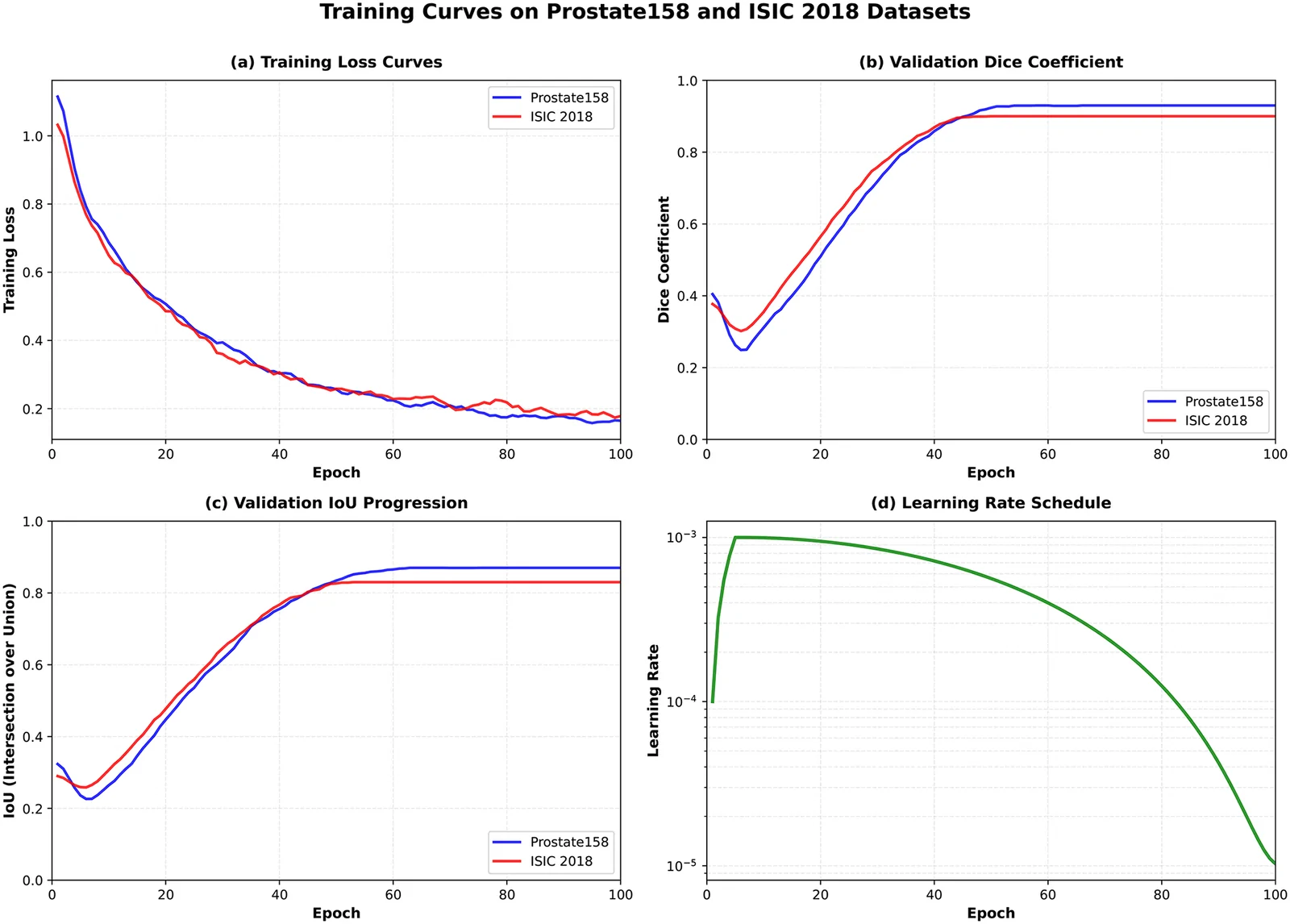
Training curves. The curves illustrate model optimization during training and are used to assess convergence stability across epochs.

### Contextual literature comparison

For broader context, Table 8 places the local results alongside representative literature values. Because published studies may differ in data split, target definition, preprocessing, and metric aggregation, this table is interpreted as a contextual comparison rather than a direct ranking.

**Table 8:** Contextual comparison with recent literature. Entries may differ in split, target definition, preprocessing, and aggregation protocol; therefore, the comparison is descriptive rather than a direct ranking.

Method	Dataset	DSC (%)	Reference / comparability note
*Prostate Segmentation*			
U-ResNet	Prostate158	82.0	Adams et al. [37]; target and protocol may differ
3D U-Net ensemble	Prostate158	86.5	Grand Challenge 2023; source and protocol may differ
nnU-Net	Prostate158	87.1	Isensee et al. [8]; local implementation preferred
SAM-Prostate	Prostate158	88.5	Siblini et al. [26]; prompt protocol may differ
Ours	Prostate158	89.2 / 76.8 / 65.3	Local slice-based protocol; not a direct SOTA claim
*Skin Lesion Segmentation*			
DeepLabV3+	ISIC 2018	88.1	Chen et al. [21]; protocol may differ
U-Net++	ISIC 2018	91.8	Zhou et al. [20]; protocol may differ
Self-training DeepLabV3	ISIC 2018	88.0	Nowak et al. [39]; protocol may differ
Dual-stage U-Net	ISIC 2018	94.2	Rahman et al. [40]; split/protocol not identical
YOLOSAMIC	ISIC Hybrid	93.4	Liu et al. [36]; hybrid dataset not directly comparable
Ours	Internal ISIC 2018 split	94.8	Local split; not a direct SOTA claim

## Discussion

### Key findings

The results support a focused conclusion: under the implemented local protocols, adding hybrid point prompts, PEFT-based SAM adaptation, and post-processing to a YOLOv8 + SAM box-prompt baseline yields higher point estimates on Prostate158 and the internal ISIC 2018 split. These findings suggest that prompt quality and medical-domain adaptation can be useful within an automatic detection–segmentation cascade. However, the current evidence is interpreted as two independently trained benchmark evaluations rather than proof of broad cross-domain generalization or clinical deployment readiness.

YOLO bounding boxes are treated as localization prompts rather than as a mechanism that directly reduces the computational burden of the SAM image encoder. Standard SAM computes a full-image embedding before prompt-conditioned mask decoding; therefore, any claimed reduction in image-encoder computation requires an explicitly implemented and benchmarked ROI-cropping strategy before image encoding.

### Comparison with existing YOLO-SAM and SAM-prompt methods

This study compares the present additions with related work item by item rather than claiming novelty for the overall cascade in Table 9. Compared with Pandey et al. [15], which used YOLOv8 boxes to drive SAM, the present framework evaluates additional positive and negative point prompts generated from detector outputs. Compared with prompt-engineering work such as Siblini et al. [26], the present framework emphasizes automatic prompt generation rather than manually specified or oracle prompts. Compared with YOLOSAMIC [36], the present study evaluates both prostate MRI and dermoscopy data, but this is described as multi-dataset applicability rather than direct superiority because protocols and datasets are not necessarily identical.

**Table 9:** Itemized comparison with closely related work. The table highlights the specific methodological differences considered in this study.

Method	YOLO-to-SAM cascade	Hybrid point prompts	SAM PEFT	Protocol note
Pandey et al. [15]	Yes	Limited / box-focused	Not central	Multimodal setting; protocol differs
Siblini et al. [26]	Not the main focus	Prompt strategy studied	Not central	Prompt protocol may include oracle/manual prompts
YOLOSAMIC [36]	Yes	Task-specific	Not directly comparable	Hybrid ISIC setting; protocol differs
Ours	Yes	Box + positive + negative points	Prompt encoder and mask decoder	Local slice-based / internal split protocols

### Clinical significance

The clinical interpretation is deliberately conservative. The current study does not include a prospective clinical trial, reader study, external prostate validation cohort, smartphone deployment experiment, diagnostic risk prediction model, or workflow evaluation. Therefore, the results indicate potential utility for reducing manual contouring burden and supporting quantitative image analysis, but they do not demonstrate clinical effectiveness, diagnostic benefit, or improved patient outcomes.

For prostate MRI, the reported 0.3-second inference time refers to single-slice processing on an A100 GPU under the stated implementation. Because the current framework processes 2D slices, the total per-examination runtime depends on the number of slices, preprocessing overhead, data transfer, and post-processing. It is therefore reported separately as per-slice and per-volume runtime. For dermoscopy, the ISIC 2018 results suggest that the method may be relevant to teledermatology image analysis, but smartphone screening, remote triage, and early-detection claims require dedicated validation and are described only as future application hypotheses.

### Limitations

Despite the experimental results achieved in this study, several limitations warrant candid acknowledgment. **First**, the Prostate158 experiment is based on 2D slice processing, whereas prostate MRI is intrinsically volumetric. The present results do not report 3D Dice, inter-slice consistency, Surface Dice, or 3D HD95. This limits direct clinical interpretation for volumetric measurement and treatment planning. **Second,** the prostate test set contains only 19 cases, so single point estimates are insufficient; per-case distributions, confidence intervals, paired tests, and multiple training seeds are required. **Third**, the detector is a possible failure point. If YOLO misses a small PCa lesion, the SAM stage is not triggered. Detector recall, false-negative counts, and unconditional end-to-end segmentation scores must therefore be reported together with conditional segmentation scores after successful localization. **Fourth**, the ISIC 2018 experiment uses an internal 70/15/15 split rather than the official challenge test set. As a result, comparisons with Rahman et al. [40], Liu et al. [36], or other literature values are not like-for-like unless identical data, split, preprocessing, target definition, and aggregation protocol are verified. **Fifth**, the validity of automatically generated positive prompts is not guaranteed for irregular or concave targets. The proportion of invalid positive points and the sensitivity to prompt randomness remain important quantities for error analysis. **Finally**, current clinical claims are limited by the absence of external validation, prospective testing, reader studies, and deployment experiments.

### Future directions

Addressing the limitations identified above, we propose the following directions for future work. **First**, future work should extend the framework toward three-dimensional volumetric segmentation. A 3D detector or volumetric region proposal module could be combined with 3D SAM variants such as SAM-Med3D [28] or MedLSAM [41] to incorporate inter-slice consistency and support clinically meaningful 3D measurements. **Second**, future studies should provide protocol-matched external validation, multiple training seeds, case-level uncertainty intervals, and paired statistical tests. **Third**, future work should explicitly model detector uncertainty and missed-detection propagation, for example by allowing fallback prompts, uncertainty-aware manual-check flags, or clinician correction of failed localizations. **Finally**, prospective reader studies and workflow evaluations are necessary before clinical efficiency or telemedicine claims can be made.

## Conclusion

This study presents a slice-based automatic YOLO-SAM hybrid prompting framework for medical image segmentation. The framework combines YOLOv8-based localization, hybrid box/point prompt generation, parameter-efficient SAM fine-tuning, and lightweight post-processing. Under the implemented Prostate158 and internal ISIC 2018 protocols, the method achieves competitive point estimates compared with locally implemented baselines, with the largest observed improvement in the prostate cancer lesion segmentation task. The manuscript avoids unsupported state-of-the-art and clinical-deployment claims, clarifies prompt-source fairness, specifies the slice-based evaluation protocol, corrects the ablation interpretation, and identifies the detector metrics, uncertainty estimates, statistical tests, and data-release items required for a fully auditable experimental record. Future work will focus on 3D volumetric extension, protocol-matched external validation, uncertainty-aware detector–segmentor integration, and prospective clinical evaluation.
